# Peripheral inflammatory markers in amnestic mild cognitive impairment

**DOI:** 10.1002/gps.3988

**Published:** 2013-07-15

**Authors:** Salman Karim, Steve Hopkins, Nitin Purandare, Jackie Crowther, Julie Morris, Pippa Tyrrell, Alistair Burns

**Affiliations:** 1 Division of Psychiatry, University of ManchesterManchester, UK; 2 Brain Injury Research Group, Salford Royal Foundation TrustManchester, UK; 3 Psychiatry, University of Exeter Medical SchoolExeter, UK; 4 Institute of Psychology, Health and Society, University of LiverpoolLiverpool, UK; 5 Department of Medical Statistics, University Hospital of South ManchesterManchester, UK; 6 Stroke Medicine, University of ManchesterManchester, UK; 7 Division of Psychiatry, University of ManchesterManchester, UK

**Keywords:** CRP, IL6, inflammatory markers, mild cognitive impairment

## Abstract

**Objective:**

To prospectively monitor plasma inflammatory marker concentrations in peripheral blood, over 12 months, in subjects with amnestic mild cognitive impairment (MCI), and to determine the relationship between peripheral inflammatory markers and cognitive decline.

**Methods:**

Seventy patients with amnestic MCI were recruited from two sites providing specialist memory assessment services in Manchester. The baseline assessment included physical examination, neuro-psychological testing and venous blood samples for C-reactive protein (CRP) and interleukin 6 (IL-6) concentrations. Sixty two participants were followed up after 12 months and the assessments were repeated.

**Results:**

Data analysis revealed a significant rise in CRP, but not IL-6 concentrations over 12 months, which was not confounded by demographic variables. The neuro-psychological test scores had no association with CRP or IL-6 concentrations at baseline or 12 months follow-up.

**Conclusion:**

This study adopted the unique approach of prospectively investigating peripheral inflammatory markers in a cohort with amnestic MCI. A significant rise in CRP concentrations over 12 months, but lack of significant association with cognition, provide no evidence for a relationship between systemic inflammation and cognitive decline in amnestic MCI.

## Introduction

There has been accumulating evidence of the role of inflammation in the aetiology of Alzheimer’s disease (AD) (Eikelenboom *et al*., 2002; McGeer and McGeer, 2007; Tan and Seshadri, 2010). Population-based studies suggest that long term use of non-steroidal anti-inflammatory drugs can be protective and delay the onset of AD (De Craen *et al*., 2005; Vlad *et al*., 2008). However, a number of clinical trials in people with established cognitive decline have failed to demonstrate benefit of treatment with anti-inflammatory drugs (McGeer and McGeer, 2007). A possible explanation for these apparently conflicting results is that these studies did not involve intervention at a sufficiently early stage (Duthie *et al*., 2011) thus raising the importance of early detection using biological markers of inflammation. An understanding of the relationship between inflammatory processes and the development of AD is likely to be fundamental to establish future therapeutic opportunities (Cuello, 2011).

Mild cognitive impairment (MCI) represents the intermediate stage between normal ageing and dementia (Petersen, 2011) and can be broadly divided into amnestic and non-amnestic MCI, depending upon the presenting cognitive difficulties. People with amnestic MCI present with prominent memory difficulties and are at high risk of developing AD, with a conversion rate of 16% per year as compared with 1%–2% in general population (Petersen *et al*., 2005). Identifying those most at risk of conversion could provide the opportunity to develop early detection and disease modifying strategies for AD.

Both innate and cell-mediated immune mechanisms have been proposed to be involved in the pathogenesis of AD (Mattson, 2004). Activated microglia congregate around amyloid plaques and degenerating neurons to release a number of inflammatory mediators, including cytokines, classical complement pathway components and chemokines (Moore, 2002; Wyss-Coray, 2005; Steinman, 2008). This suggests a low grade inflammatory process and the possibility that inflammatory markers could be used to aid the diagnosis or progression of AD. The association between elevated peripheral inflammatory marker concentrations including C-reactive protein (CRP) and interleukin-6 (IL-6), and cognitive decline has been reported in cross-sectional studies of the general population (Oztürk *et al*., 2007; Alley *et al*., 2008) and in people with AD and MCI (Guerreiro *et al*., 2007; Magaki *et al*., 2007; Roberts *et al*., 2009). Similarly, the presence of activated microglia in the brain can provide evidence of central inflammation which can be visualized *in vivo* by using positron emission tomography with the [11C](R)-PK11195 ligand (Banati, 2002). Increased [11C](R)-PK11195 binding has been reported in AD (Edison *et al*., 2008) and MCI (Okello *et al*., 2009).

We designed a study to evaluate the changes in peripheral inflammatory markers, over time, in subjects with amnestic MCI and to determine the relationship between peripheral inflammatory markers and cognitive decline.

## Methods

Potential participants were identified and approached from two services including the Memory Clinic at Wythenshawe Hospital in South Manchester and old age psychiatry department at Stepping Hill Hospital in Stockport. Those recruited underwent a comprehensive assessment process, including history taking, neurological examination, cognitive assessment, routine blood tests and CT brain scans to exclude vascular and structural pathology. A diagnosis of amnestic MCI was established, using Petersen’s criteria (Petersen *et al*., 2005), by a multidisciplinary team including a consultant old age psychiatrist, psychologist and nurses. All participants were assessed for the possibility of cerebrovascular disease as a cause of cognitive decline in the multi-disciplinary assessment. People with a history of stroke or transient ischemic attack in their medical records were not excluded if, at the time they occurred, these events were not associated with cognitive decline and if there was no evidence of significant cerebrovascular disease on CT scans. People with other vascular risk factors including hypertension, diabetes, dyslipidemia, atrial fibrillation and coronary artery disease were also not excluded. All the participants were approached for follow-up after 12 months.

The baseline and follow-up assessments included demographic and clinical information, physical examination, infection screening questionnaire and neuropsychological assessments. The neuropsychological assessments included Mini Mental State Examination (MMSE; Folstein *et al*., 1975) as the primary outcome measure of cognition along with Cambridge Cognitive Examination-Revised (CAMCOG-R; Roth *et al*., 1999); National Adult Reading Test (NART; Blair and Spreen, 1989); Free and Cued Recall Selective Reminding Test (FCRSRT; Grober and Buschke, 1987); Verbal fluency test (FAS; Spreen and Strauss, 1998); and Cognitive Estimation Test (CET; Axelrod and Mills, 1994) as secondary measures.

Clinical information included vascular risk factors, history of infections and current list of medication. Physical examination included height, weight, pulse and blood pressure.

Venous blood (15 ml) was taken between 0900 h and 1230 h for the plasma measurement of IL-6 [measured using a sandwich enzyme-linked immunosorbent assay (ELISA)] and CRP (using a high sensitivity competitive enzyme-linked immuno assay) and Apolipoprotein E status (APoE) measured by polymerase chain reaction (PCR).

An infection screen questionnaire (Appendix 1) was administered. In patients with positive infection, screen blood sampling was delayed by 2 weeks or until the infection was settled. If the CRP was raised above 3 mg/l, a second blood sample was taken, for repeat CRP measurement, after an interval of at least 6 weeks. The lower of the two readings was used for the data analysis. This resulted in 16 baseline and 16 follow-up CRP measurements being repeated. The IL-6 measurements were undertaken using the same plasma sample as that used for CRP.

Written, informed consent for participation, follow-up assessments and genetic testing was obtained by the researchers at the time of recruitment. The study protocol was approved by the local research ethics committee (National Research Ethics Committee North West – Greater Manchester North).

## Statistical analysis

A power calculation established that with 70 participants correlations of at least 0.33 between inflammatory markers and cognitive decline would be detectable with 80% power. The data was analysed using spss version 16.0 (IBM, Armonk, NY, USA). Values of CRP and IL-6 had a skewed distribution and required a natural logarithm transformation to approximate a normal distribution. Simple paired *t*-tests were used to assess changes in measures over the 12 month follow-up. Associations between inflammatory markers and cognitive decline were investigated using Pearson’s correlation coefficients. Regression analyses were used to assess the relationship between inflammatory markers concentrations and cognition controlling for the confounding role of demographic/baseline features.

## Results

We approached 120 potential participants, 70 were recruited and 62 were followed up after 12 months. The demographics of participants are presented in Table 1. Full blood counts were within normal range. Mean baseline concentrations of CRP and IL-6 are shown in Table 2. The mean CRP concentrations increased significantly over 12 months, but there was no statistically significant change in IL-6. The cognitive test results (Table 3) showed that there was no significant change in either the primary or secondary measures over the follow-up period. There was also no statistically significant association between the change in IL-6 or CRP and any of the cognitive measures (all correlation values were less than 0.25). Multiple regression analysis revealed that this lack of association was not confounded by demographic/baseline features including age, sex, smoking status, body mass index, systolic blood pressure, history of stroke, transient ischemic attack or diabetes.

**Table 1:** Demographics of MCI patients

Characteristic	*N* (%); Mean [range]
Male	42 (60)
Age (years)	69.8 [68–72]
Ethnicity (white)	59 (84)
Ethnicity (other)	11 (16)
Vascular risk factors:	
Smokers (current)	12 (17)
Smokers (ex)	35 (50)
Hypertension	27 (39)
Diabetes mellitus	7 (10)
Stroke	15 (21)
Dyslipidemia	39 (56)
Atrial fibrillation	13 (19)
Coronary artery disease	17 (24)
Peripheral vascular disease	8 (11)
APoE positive	37 (53)
Aspirin and statin	38 (54)
NSAIDs*	26 (37)
Height (meters)	1.67 [1.64–1.69]
Weight (kilograms)	75.2 [71–79]
Waist (inches)	37.2 [36–38.5]
Body mass index	26.8 [26–28]
Systolic blood pressure (mm of mercury)	141 [136–148]

MCI, mild cognitive impairment; APoE, apolipoprotein E status.

* Non-steroidal anti-inflammatory drugs other than aspirin.

**Table 2:** Inflammatory marker concentrations of MCI patients over 12 months

	Geometric mean values (range)			
Inflammatory Marker	Baseline	1 year	Ratio 1 year/baseline (95% CI)	Significance
IL-6 (pg/mL)	2.3 (≤0.7, 21.3)	2.4 (≤0.7, 24.9)	1.0 (0.8, 1.2)	*p* = 0.78
CRP (mg/L)	1.5 (0.1, 9.9)	1.8 (0.4, 20.5)	1.2 (1.0, 1.4)	*p* = 0.020

MCI, mild cognitive impairment; CI, confidence interval; IL-6, interleukin-6; CRP, C-reactive protein.

**Table 3:** Cognitive test results of MCI patients over 12 months

	Mean (SD) values			
	Baseline	1 year	Difference (95% CI)	Significance
MMSE*	26.9 (2.3)	26.3 (3.3)	−0.5 (−1.2, 0.1)	*p* = 0.10
FAS**	37.3 (11.0)	38.8 (12.4)	1.4 (−0.8, 3.7)	*p* = 0.21
CAM Cog**	90.4 (7.4)	89.0 (11.1)	−1.3 (−3.0, 0.3)	*p* = 0.10
Cognitive estimates**	6.3 (4.1)	7.0 (4.4)	0.8 (−0.2, 1.7)	*p* = 0.13
BUSCHKE**	79.7 (22.4)	80.2 (24.1)	0.5 (−3.9, 4.9)	*p* = 0.82

SD, standard deviation; CI, confidence interval; MMSE, Mini Mental State Examination; FAS, verbal fluency test; CAM Cog, Cambridge cognitive examination.

* Primary outcome measure.

** Secondary outcome measure.

As more than half of the cohort (*n* = 43) was on anti-inflammatory medication including aspirin and statins, the possible relationship between CRP and IL-6 concentrations and cognition were explored between the two groups. We detected no significant differences in baseline measures and change over 1 year of either CRP and IL-6 concentrations or cognition.

We also looked at the possible differences in changes of inflammatory marker concentrations and cognitive measures over 12 months between the APoE positive and negative patients. No significant difference was detected in changes in either IL-6 or CRP concentrations and cognitive measures between the two groups. Two of 62 patients were diagnosed to have converted to AD over the 12 month follow-up period.

## Discussion

The results of our study show that plasma CRP, but not IL-6, concentrations rose over the 1 year period. No significant correlation between change in CRP or IL-6 concentrations and cognitive decline was detected.

Studies exploring the link between peripheral inflammatory markers and cognitive decline have reported conflicting results. A number of studies reported an association between raised CRP and IL-6 concentrations and cognitive decline (Yaffe *et al*., 2003; Oztürk *et al*., 2007; Alley *et al*., 2008), whereas others provided negative results (Dik *et al*., 2005; Weuve *et al*., 2006). One study, looking at the inflammatory marker concentrations in MCI and AD subjects, reported significantly raised concentrations of CRP in peripheral blood in MCI subjects as compared with AD (Schuitemaker *et al*., 2009), yet another showed raised concentrations of IL-6 in AD as compared with MCI (Bermejo *et al*., 2008). In these studies, however, the concept of MCI was applied relatively loosely, not using specific criteria. There have been very few studies on amnestic MCI. Roberts *et al*. (2009), in a population-based sample of older people, studied amnestic and non-amnestic MCI patients and reported significant association between CRP concentrations and non-amnestic MCI, but they did not find an association between CRP, IL-6 and cognitive decline in amnestic MCI. They also reported that in their sample, the APOE-4 positive cases had lower CRP concentrations as compared with non-amnestic MCI group, a finding that has been replicated by other studies (Eiriksdottir *et al*., 2006; Laurin *et al*., 2009). In a recent cross-sectional study, Zhao *et al*. (2012) found that concentrations of IL-6 were significantly higher in an amnestic MCI group than in age matched healthy controls. In their cohort, APOE4 positive participants had significantly lower IL-6 concentrations as compared with APOE4 negative participants. They also detected a weak, but significant, negative correlation between IL-6 and cognitive performance. They concluded that IL-6, although not useful alone, has potential, in combination with other biomarkers, to support early diagnosis of amnestic MCI, because of its association with the progression of cognitive impairment.

None of these studies, to our knowledge, made rigorous attempts to avoid changes in inflammatory marker concentrations due to acute events such as infection. The strengths of our study include the prospective design, detailed neuropsychological testing, use of specific criteria for amnestic MCI and protocol for minimizing the possibility of raised inflammatory marker concentrations due to infection.

The possibility of the ageing process on its own resulting in the rise of inflammatory marker levels over 12 months or in combination with chronic physical health problems (Table 1) has to be considered as reported by Godbout *et al*. (2004). Although every effort was made to avoid erroneously raised inflammatory marker levels due to acute events, it is possible that these factors could have influenced the results.

Depression in general and major depressive disorder in particular has been linked with acute phase inflammatory mediators including CRP (Irwin and Miller, 2007; Raison *et al*., 2006). In our study, although the clinical diagnostic process of amnestic MCI included the exclusion of mood disorders, the participants were not assessed for the possibility of mood disorders as part of the assessment process. The likelihood of the participants developing major depressive disorder however is minimal as they remained under the care of mental health services. It is possible that undetected mild to moderate mood changes during the 1 year follow-up period could have influenced the results.

In our cohort, the conversion rate to AD was lower (2 out of 62 participants converted to AD over 12 months) than the expectation of around nine conversions on the basis of Petersen *et al*. (2006) figures of 16% conversion rate. One possible explanation could be the small size of the cohort, although it is also possible that those lost to follow-up might have converted to AD and moved from their home address.

Our study did not reveal an association between peripheral inflammatory marker concentrations and cognitive decline over 12 months. This result could be due to a relatively small sample size and absence of significant changes in all cognitive measures in the cohort. It is possible that a larger sample size and longer follow-up period may have revealed a significant association between cognitive decline and inflammatory marker concentrations, but the present data certainly indicates that this relationship is likely to be relatively weak in individuals at this stage of cognitive decline. The rise in the CRP concentrations over the follow-up period could be unrelated to AD type neurodegenerative processes and linked to other factors, such as vascular disease and ageing (Tan *et al*., 2007).

## Conclusion

On the basis of the accumulating scientific evidence of the role of inflammatory processed in MCI and AD, over two decades, this study adopted the unique approach of prospectively investigating peripheral inflammatory markers in a group of participants with amnestic MCI. The significant rise in CRP concentrations over 12 months, but lack of significant association with cognitive decline, provides no evidence for a relationship between systemic inflammation and cognitive impairment in amnestic MCI.

## Dedication

We would like to dedicate this paper to the fond memory of our colleague Prof. Purandari who is sadly no longer amongst us.

## Key points

- Relationship between cognitive decline and a rise in inflammatory marker levels in peripheral blood has been reported in a number of studies.
- The relationship between peripheral markers of inflammation and cognitive decline in general and Alzheimer’s disease in particular appears to be complex and influenced by a number of factors.
- Our prospective study of people with amnestic mild cognitive impairment did not reveal any relationship between CRP and IL6 levels in peripheral blood and cognitive decline.

## Appendix 1: Infection screen

**Table:**

A cold or cold-like symptoms?	□
Cough?	□
Cough productive of sputum?	□
Sore throat?	□
Episodes of high temperature?	□
Episodes of shivering?	□
Discharge from the ears?	□
Discharge from eyes?	□
Stomach upset such as vomiting and/or diarrhoea?	□
Discomfort or burning when passing urine?	□
Dental/oral infection?	□
Skin rashes/boils/blisters/abscesses/wounds?	□
Any other infection that you are aware of?	□
